# Protein Synthesis Determined from Non-Radioactive Phenylalanine Incorporated by Antarctic Fish

**DOI:** 10.3390/metabo13030338

**Published:** 2023-02-24

**Authors:** Nina Krebs, Jan Tebben, Christian Bock, Felix C. Mark, Magnus Lucassen, Gisela Lannig, Hans-Otto Pörtner

**Affiliations:** 1 Department of Integrative Ecophysiology, Alfred Wegener Institute, Helmholtz Centre for Polar and Marine Research, Am Handelshafen 12, 27570 Bremerhaven, Germany; 2 Department of Ecological Chemistry, Alfred Wegener Institute, Helmholtz Centre for Polar and Marine Research, Am Handelshafen 12, 27570 Bremerhaven, Germany

**Keywords:** polar fish, isotopic labelling, 13C-phenylalanine, slow metabolism, somatic growth

## Abstract

Direct measurements of temperature-dependent weight gains are experimentally challenging and time-consuming in long-lived/slow-growing organisms such as Antarctic fish. Here, we reassess methodology to quantify the in vivo protein synthesis rate from amino acids, as a key component of growth. We tested whether it is possible to avoid hazardous radioactive materials and whether the analytical pathway chosen is robust against analytical errors. In the eelpout, *Pachycara brachycephalum*, ^13^C_9_H_11_^15^N_1_O_2_ phenylalanine was injected intraperitoneally and muscle tissue was sampled before injection and at 1.5 h time intervals up to 6 h thereafter. The incorporation of ^13^C^15^N-labeled-phenylalanine into muscle was monitored by quantification of bound and free phenylalanine through liquid chromatography–mass spectrometry. We found an increase in the pool of labeled, free phenylalanine in the cytosolic fraction that leveled off after 4.5 h. The labeled phenylalanine bound in the proteins increased linearly over time. The resulting protein synthesis rate (Ks) for *P. brachycephalum* was as low as 0.049 ± 0.021% day^−1^. This value and its variability were in good agreement with literature data obtained from studies using radioactive labels, indicating that this methodology is well suited for characterizing growth in polar fish under in situ conditions in remote areas or on research vessels.

## 1. Introduction

Protein synthesis rates are a key indicator for organismal responses to environmental change [1]. Reliable and accurate methodologies are a prerequisite for analyzing protein synthesis rates in different species and their tissues [2]. Protein synthesis is one of the most energetically costly processes in cell metabolism, amounting to up to 42% of the overall energy expenditure of ectotherms [2]. Protein synthesis in fish white muscle contributes up to 79% to growth performance [3]. Furthermore, the rate of protein synthesis characterizes species and their tissues from various environmental regions as well as their responses to factors such as seasonal changes, temperature and food quantity [4,5,6,7]. For example, protein synthesis quantified as Ks (fraction of total protein synthesized per day) was reported to be higher in temperate zone fish species (4.4% day^−1^ in white muscle tissue of young rainbow trout [8]) than in species found in the tropics (about 0.17 to 0.07% day^−1^ [9]) or in polar regions (0.04 to 0.25% day^−1^ [10,11]). Fractional protein synthesis rates of rainbow trout varied by tissue type, with the highest rates in gills (up to 9% day^−1^) and the lowest in white muscle (0.5% day^−1^ [3]). The rate of protein synthesis correlates with tissue oxygen demand [12].

Protein synthesis can be measured in vitro or in vivo. In vitro measurements utilize isolated cells or tissues cultured in a “Ringer” medium that contains labeled amino acids. The in vitro incorporation of labeled amino acids reflects the cellular protein synthesis rate and the study of isolated cells excludes possible (and important) influences on the whole animal and its metabolism [12,13]. In vivo measurements mainly derive protein synthesis rates from constant infusion, stochastic endpoint studies or the flooding dose technique [2]. A constant infusion provides a constant supply of labeled tracers via venous cannulation eliminating the risk of fluctuating concentrations [11,14]. This method, however, is less applicable for mobile animals that require anesthesia to stop them from removing the venous cannulation. It needs to be considered that anesthesia by itself may cause artefactual changes in the rate of protein synthesis [15]. For the stochastic endpoint study animals are fed with labeled tracers, such as proteinogenic amino acids, for days or weeks and the difference between intake and excretion reflects the protein synthesis rate. It integrates the protein synthesis rate across all tissues and it is non-invasive [16,17]. A faster and more specific approach is the injection of a flooding dose of labeled tracers as developed by Garlick et al. [18]. Here, organisms are provided with an intravenous or intraperitoneal injection of a large dose of a tracer in combination with a non-labeled tracer which floods all tissues within a short period of time until terminal sampling of the tissues [3,7,9,18,19,20,21]. The sampling is generally undertaken over several time steps and is dependent on several assumptions listed in Fraser and Rogers, 2007 [2]:

1. “The intracellular free-pool specific radioactivities are elevated and stable during the protein synthesis measurement”
2. “Incorporation of radiolabeled” (or isotopically labeled) “amino acid into the bound protein pool should be linear and significant over the time course of protein synthesis”
3. “The injected amino acid should flood the plasma, intracellular, extracellular and aminoacyl tRNA pools. Typically, successful flooding doses will elevate intracellular concentrations of the amino acid by about four- to tenfold.”
4. “Injecting the amino acid should not result in an elevation of the rate of protein synthesis”

In contrast to amino acids such as leucine [22], the essential amino acid phenylalanine (Phe) is commonly used as a tracer because it does not influence protein synthesis [18] when injected in high amounts, fulfilling the fourth criterion named by Fraser and Rogers (2007) [2]. Phe, however, can have negative effects on the brain when being enriched over a longer period of time. Findings in mammals indicate that, under such conditions, phenylalanine hydroxylase converts phenylalanine into tyrosine [23]. While working with radioactive tracers it is not possible to differentiate between radioactively labeled phenylalanine and tyrosine. Therefore, Garlick et al. (1980) included large amounts of unlabeled phenylalanine or included other amino acids in the injection. This should minimize any effect of unwanted radioactively labeled tyrosine [18].

Until today, most studies have used radioactively labeled Phe to calculate protein synthesis rates [7,12,13,18,24,25]. However, the background content of Phe varies as every organism has a different composition of amino acids dependent on environmental factors and metabolism [26]. In Antarctic eelpout, the level of protein-bound Phe is 0.74% of white muscle tissue and 0.42% in gills [13]. Antarctic eelpout are benthic Antarctic fish with a low metabolic rate [27]. Tuna, for comparison, live in warmer waters and have higher swimming rates which are reflected in a very high metabolic rate. Their Phe content amounts to 1–2.5% of the whole animal with some variability during development [28].

Radioactive labeling supports very sensitive and accurate measurements especially needed for animals with low protein turnover rates such as Antarctic species [10,11]. However, radioactivity requires significant radiation protection infrastructure, and is mainly used in laboratories, as field studies are logistically challenging (for a recent review see Cresswell et al. 2020 [29]). In recent years, substances labeled with non-radioactive isotopes were used instead of radioisotopes to limit the use of hazardous compounds [19,30,31,32,33,34,35]. Isotopically labeled tracers bound into proteins as well as unbound in the cytosolic fraction can be quantified with analytical methods such as NMR spectroscopy [30,35] and mass spectrometry [5,9,19,21]. These techniques have the advantage that the labeled isotope as well as the natural (unlabeled) metabolite can be analyzed within the same sample, thereby increasing the accuracy of the labeled and unlabeled fractions against the background of the existing (unknown) pool of Phe.

The aim of the study was to measure the protein synthesis rate of Ks in vivo in an ectothermic polar fish with very low metabolism and growth rates. Using the Antarctic eelpout (*Pachycara brachycephalum*) as a model organism, we applied an intraperitoneal injection of a flooding dose of Phe labeled with a stable isotope in fish. For the detection of labeled and unlabeled Phe, we used liquid chromatography with high-resolution tandem mass spectrometry (LC-HRMS/MS). By using LC-HRMS/MS, we were able to simultaneously quantify labeled and natural, unlabeled phenylalanine in both the cytosol and the protein-bound fraction, which allowed us to calculate net protein synthesis in vivo and to non-radioactively measure very small changes of labeled phenylalanine in the white muscle. We suggest that this method can be easily deployed on ships and/or in remote locations such as stations in Antarctica, providing opportunities to study, for example, the effects of climate change on polar ecosystems and organisms directly on site.

## 2. Materials and Methods

The Antarctic eelpout, *Pachycara brachycephalum* (Pappenheim, 1912), were caught in Admiralty Bay, King George Island (62°11′ S, 58°20′ W), Antarctica, by baited fish traps between 430 and 530 m depth on RV Polarstern expedition PS112 in March 2018. The fish traps were recovered from the seafloor after 52 h. On board, the fish were kept at 0 °C for the duration of their transport to the Alfred Wegener Institute (AWI), Bremerhaven, Germany. At the end of the cruise, more than 99.5% survived. At AWI, the fish were kept in well-aerated, re-circulating seawater at 0.0 ± 0.5 °C and 34 ± 1 practical salinity units (PSU) under a 12:12 light:dark cycle that remained unchanged during the experiments. The fish were fed with frozen blue mussel, *Mytilus edulis* (Erdtmann, Salzwedel, Germany) once a week. All selected animals were not fed for 5–7 days before the start of the experiments.

### 2.1. Chemicals and Instruments

The chemicals used for the preparation of the saline injection buffer were all HPLC grade (Carl Roth, Karlsruhe, Germany; Sigma-Aldrich, St. Louis, MO, USA or VWR, Radnor, PA, USA). Labeled phenylalanine (^13^C_9_H_11_^15^N_1_O_2_, 98% purity) was purchased from Sigma-Aldrich. Methanol (MeOH) and formic acid (FA) were HPLC grade (Roth, VWR). Hydrochloric acid was purchased from Merck (Merck KGaA, Darmstadt, Germany). Chromabond C18 Hydra 1 mL 100 mg SPE cartridges (Macherey-Nagel, Düren, Germany) were used for the solid phase extraction. Ultrapure water was generated with an Arium Pro system (Sartorius, Göttingen, Germany). A vacuum centrifuge (Speedvac, Thermo Fisher Scientific, Waltham, MA, USA) and lyophilizer (Martin Christ Gefriertrocknung GmbH, Osterode am Harz, Germany) were used to dry the samples.

### 2.2. Intraperitoneal Injection and Tissue Collection

Antarctic eelpout (total length: 22.6 ± 2.5 cm, total weight: 41.3 ± 12.8 g, n = 30) were first weighed and the conscious fish then immediately injected with 0.7 mL/100 g body weight of 75 mM of ^13^C_9_H_11_^15^N_1_O_2_ phenylalanine in PBS buffer (pH 7.4, 4 °C) into the abdomen by use of a 1 mL syringe and a 0.40 × 20 mm cannula (Braun Melsungen AG, Melsungen, Germany). Shortly after injection, there was no visible stress response by the animal. To measure the protein synthesis response after phenylalanine injection, fish were kept at 0 °C and sampled at four time points after injection: 1.5, 3, 4.5 and 6 h (and compared against a control group). At each time point, six randomly selected fish were sacrificed. Fish were first stunned with a blow to the head and then killed by cutting the spinal cord closely behind the head. White muscle tissue was then collected and quickly frozen in liquid nitrogen and stored at −80 °C until further use. Handling, injection and killing of the fish were conducted in compliance with German legislation and in line with the recommendations of the American Veterinary Medical Association (AVMA). The work was approved by the German authority (Freie Hansestadt Bremen, reference number 160; 500-427-103-7/2018-1-5)

### 2.3. Methanol Chloroform Extraction

White muscle tissue was extracted according to Wu et al. [36]. Briefly, 50 mg of the frozen muscle tissue was homogenized in 400 µL of methanol (MeOH) and 125 µL of ultrapure water in 2 circles of 20 s at 6000 rpm at 4 °C using a Precellys tissue homogenizer (Bertin Instruments, Montigny-le-Bretonneux, France). The homogenized tissue was then transferred into 1.5 mL Eppendorf tubes and 400 µL chloroform with 400 µL ultrapure water was added. This mixture was vortexed for 20 s and incubated on ice for 10 min before centrifugation for 15 min at 3000× *g* and 4 °C. The three layers comprising the upper aqueous layer, the lower chloroform layer and the precipitated protein layer in the middle were collected in separate 1.5 mL vials (Eppendorf, Hamburg, Germany). The upper aqueous layer (total volume of 800 µL) contained the cytosolic fraction with polar metabolites including the free phenylalanine that was not incorporated in proteins. The lower chloroform layer containing apolar compounds such as lipids was discarded and not used for further analyses. The protein fraction containing the phenylalanine incorporated in proteins was washed twice to remove any residual unbound phenylalanine by adding 1 mL of MeOH followed by 20 s vortexing (Vortex mixer, Scientific Industries, Bohemia, NY, USA) and centrifugation (Eppendorf) for 3 min at 13,000× *g*. Both supernatants were collected separately. The washed protein pellet was dried in a vacuum centrifuge at 25 °C overnight. The protein pellet was then hydrolyzed with 100 µL 6M HCl/mg protein pellet for 24 h at 99 °C while shaking at 600 rpm (Thermomixer comfort, Eppendorf). The supernatant was collected by centrifugation (10,000 rpm for 3 min), frozen and lyophilized for 12 h. The dry hydrolysate was dissolved by vortexing for 20 s in 1 mL MeOH and desalted by application to a solid phase extraction (SPE). The aqueous, cytosolic layer was diluted 1:10 with MeOH and also applied to SPE without further dilution resulting in samples that contained the equivalent of 0.1 mL per 1 mL.

### 2.4. Phenylalanine Quantification

Liquid chromatography high-resolution mass spectrometry (LC-HRMS/MS) analysis was performed with a Vanquish UPLC system coupled to a Q-Exactive Plus mass spectrometer, using a heated electrospray ionization source (all Thermo Fisher Scientific). Separation was performed on a C18 column (C18 BEH, 100 × 2 mm, 1.7 μm particle size, Waters, equipped with guard-column). Positive Ion Calibration Solution (Pierce, Thermo Fisher Scientific) was used for the calibration of the instrument. A blank as well as a quality control standard was injected every five samples to check the instrument’s drift and carry-over. A binary solvent gradient was used with a flow rate of 0.35 mL per min on a C18 column (C18 BEH, 100 × 2 mm, 1.7 µm particle size, Waters, equipped with guard-column) at 32 °C, with solvent A = 0.1% formic acid in ultrapure water and solvent B = 0.1% formic acid in methanol. The gradient program was as follows: T0 min: B = 2%, T0.1 min B = 2%, T3.9 min: B = 99%, T4.5 min: B = 99%; T4.7 min: B = 2%. The column was equilibrated for 0.5 min between samples. MS spectra were acquired in full scan mode or data independent (DIA) mode. Full scans were acquired with a resolution of 35,000 (fifty percent of the maximum peak height (FWHM), *m*/*z* 200), a scan range of 120 to 250 *m*/*z*, automatic gain control (AGC) of 3 × 10^6^ and injection time (IT) of 100 ms. DIA experiments were used to quantify analytes by tandem mass spectrometry utilizing an inclusion list of the accurate masses (*m*/*z* 176.11313, *m*/*z* 166.08617, *m*/*z* 167.07617) at a resolution of 35,000 FWHM (*m*/*z* 200), NCE of 30, AGC of 2 × 10^5^ and isolation window of 1.0 *m*/*z*. The heated electrospray ionization source was set to 3.5 kV spray voltage, the aux gas to 425 °C (13) and the sheath gas to 50. The capillary temperature was set to 263 °C. The fragment ions *m*/*z* 120.0808 (^12^C_9_H_11_^15^N_1_O_2_—C_1_H_2_O_2_) and *m*/*z* 129.1047 (^13^C_9_H_11_^15^N_1_O_2_—^13^C_1_H_2_O_2_) were used for quantification with a mass tolerance of 5 ppm. Quantification was achieved with an external calibration to standard dilution series of standards prepared from ^12^C^14^N as well as the ^13^C^15^N phenylalanine ranging from 10 pg/µL to 1000 pg/µL. Calibration curves were measured every 100 samples to evaluate sensitivity changes in the instrument.

### 2.5. Calculation and Statistics

The phenylalanine concentrations in the protein hydrolysate (protein-bound) and the cytosolic fraction (free pool) were calculated using external calibration curves for each analyte. Outliers (as identified by the Inter-Quartile Range IQR) were eliminated. Ks calculation was adapted from Garlick et al., 1980 and Ks values are given as means ± standard deviation [18]. Unless otherwise stated, the data were normally (tested with Shapiro–Wilk test) and homogeneously distributed (chi-square test). Statistical differences at the level of 95% were tested by using an ordinary one-way ANOVA (analysis of variance) followed by the Tukey’s multiple comparison test as post hoc (GraphPad Prism 9).

$$\mathrm{Ks}\;(\%\;{\mathrm{day}}^{-1})=\left(\frac{\mathrm{Sb}\;\mathrm{labeled}\;\left[\frac{\mathrm{pg}}{\mu g}\right]}{\left(\mathrm{Sb}\;\mathrm{labeled}+\mathrm{Sb}\;\mathrm{unlabeled}\right)\left[\frac{\mathrm{pg}}{\mu g}\right]}\right)*\left(\frac{100}{\left(\mathrm{Sa}\;\mathrm{labeled}\left[\%\right]\right)*t\;\left(\mathrm{days}\right)}\right)*100$$

where Sb is the protein-bound pool and Sa is the free pool of phenylalanine (pg phenylalanine per µg fresh weight) and t is the time in hours. For comparability across measurements, data were calculated per day, from the time between injection and sampling in hours.

## 3. Results

**Figure 1:**
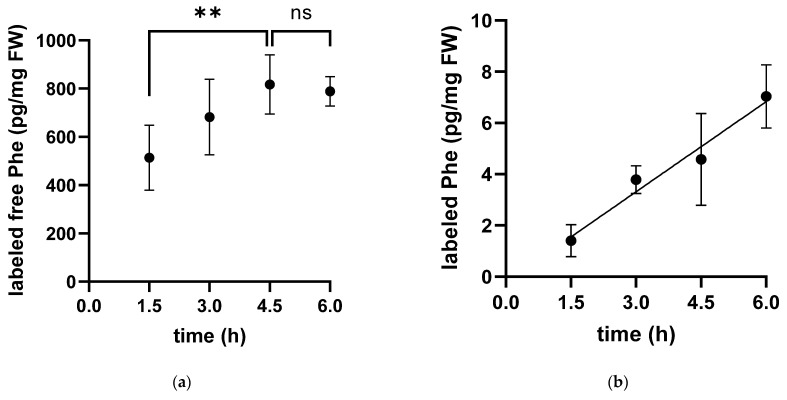
Time-dependent concentration of labeled phenylalanine in the free pool (**a**) and protein-bound (**b**) in white muscle tissue extracts of the Antarctic eelpout *Pachycara brachycephalum* kept at 0 °C. The concentration of labeled phenylalanine in the free pool (**a**) increased until reaching a plateau after 4.5 h and at a level of 900 pg/mg FW (Fresh weight) (** *p* < 0.005, ns *p >* 0.05). In contrast, the concentration of protein-bound phenylalanine (**b**) increased linearly over time up to 7 pg/mg FW (y = 1.174x − 0.2087; R2: 0.7477).

After the injection of labeled phenylalanine (Phe) into the peritoneum, the fate of the amino acid was followed over time. The concentration of labeled Phe in the free pool of the cytosolic fraction increased over time in white muscle until it reached a plateau approximately 4.5 h after injection. Accordingly, the labeled Phe was linearly incorporated into muscle protein throughout the experimental period of 4.5 h (Figure 1).

**Figure 2:**
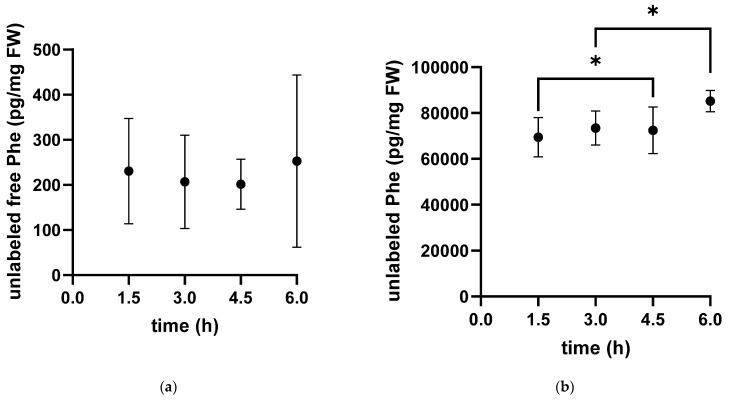
Time-dependent concentration of unlabeled phenylalanine in the free pool (**a**) and protein-bound unlabeled phenylalanine (**b**) in white muscle tissue extracts of the Antarctic eelpout *Pachycara brachycephalum* kept at 0 °C. The unlabeled free pool (**a**) decreased within the first 3 h and reached a plateau thereafter. The unlabeled bound fraction (**b**) increased over time (* *p* < 0.05).

Additionally, we measured the unlabeled content of free phenylalanine in the cytosol and of Phe bound into proteins (Figure 2). The free pool of unlabeled phenylalanine showed high variability within individuals with no clear time dependence. In contrast, the concentration of unlabeled protein-bound phenylalanine showed little variability and increased over time.

The data between 1.5 and 4.5 h meet all criteria required to calculate Ks after injection of a flooding dose. At the last sampling point (6 h), the free pool of labeled Phe did not continue to increase and the data were no longer normally distributed. Therefore, this sampling point was excluded in the following calculations. Based on these data, the calculation of protein synthesis rate Ks for each specific sampling time point yielded similar Ks values (Table 1), resulting in an average Ks of 0.049 ± 0.021% day^−1^ measured in white muscle of *Pachycara brachycephalum* at 0 °C. Additionally, the percentage of labeled phenylalanine accumulated in the pool of free Phe averaged 75.50 ± 11.07%. Both Ks and the percentage of labeled free Phe (%) remained stable between 1.5 h and 4.5 h.

## 4. Discussion

In this study, we determined the protein synthesis rate (Ks) in the white muscle of the Antarctic eelpout *Pachycara brachycephalum* in vivo after an intraperitoneal injection of a flooding dose of labeled non-radioactive phenylalanine (Phe). We analyzed the Phe content using liquid chromatography high-resolution mass spectrometry (LC-HRMS/MS) instead of labeling with radioactive isotopes. As a proof of concept, we were able to determine reliable protein synthesis rates in ectothermal organisms with slow metabolism at freezing temperatures.

To validate the reliability of the method, we quantified labeled unbound and bound Phe and thus tracked its incorporation into proteins over time. The sampling time points 1.5–4.5 h met all criteria for measuring the protein synthesis rate after injection of a flooding dose according to Fraser and Rogers, 2007 [2] (see Introduction) and are therefore considered suitable to calculate Ks. The sampling time point 6 h was excluded from the calculation of Ks because the level of labeled unbound Phe did not increase further after 6 h and the unlabeled free Phe was not normally distributed. After 6 h, no additional injected Phe is thus likely to enter the cytosol and the pre-existing Phe already present has been used to synthesize proteins or converted to tyrosine, by random the use of labeled or unlabeled Phe. Lower intake and random withdrawal of labeled and unlabeled Phe could be the reason for the high variability of the data after 6 h, resulting in a non-normal distribution. The last two criteria were met based on the experimental design and have been successfully tested in previous studies on various fish species.

The amount of labeled unbound phenylalanine varied greatly between individuals and the time of injection. On average, only 75.5 ± 11.1% of the total phenylalanine was labeled, resulting in a mean Ks value of 0.049 ± 0.021% day^−1^. This rate is about 75-times lower than previously measured in vitro in Antarctic eelpout white muscle at Ks = 3.5–3.6% day^−1^ [13], but similar to in vivo radioactive measurements in other Antarctic fish species (e.g., Smith and Haschemeyer, 1980 Ks = 0.04–0.22% day^−1^ [11]). Indeed, the authors of the in vitro study argued that the higher Ks value in *Pachycara brachycephalum* compared to other Antarctic fish could be due to the inherent differences between in vitro and in vivo measurements (see introduction). The in vitro measurement reflects the maximum capacity of protein synthesis, whereas the in vivo measurement describes a usually lower, more realistic rate. It reflects the response of the entire organism instead of individual tissues or cells [13]. In other fish species, Ks measured in vivo in white muscle varies greatly depending on biotic and abiotic factors such as age, habitat temperature, or food supply, with the highest protein synthesis rates found in well-fed juvenile rainbow trout (Ks = 4.4% day^−1^ [8]) and the lowest rates seen in starving Antarctic fish, *Trematomus hansoni* (Ks = 0.04% day^−1^ [11]). How the mentioned biotic and abiotic factors affect the protein synthesis rate of Antarctic eelpout needs to be investigated in the future.

Up to 79% of the protein synthesis rate (Ks) measured in the white muscle of rainbow trout contributed to growth [3]. On this basis, we estimated the protein growth efficiency of *P. brachycephalum* using literature data on species-specific growth rate measured over several months at 0 °C [37] and compared it with our data for protein synthesis rate (($\mathrm{Ks}/\frac{Weight\;gain\;\left(\frac{g}{d}\right)}{weigth\;\left(g\right)}*100)*100)$. Accordingly, 72–96% of Ks in white muscle contributes to growth in the Antarctic eelpout at 0 °C, an order of magnitude that confirms our approach and data. Measuring growth rates in animals with slow metabolism is time consuming, laborious and can only be performed in laboratories. However, long-term laboratory experiments of polar organisms are always associated with unpredictable risks, such as water quality degradation, that may affect results or, in the worst case, cooling failure. In addition, it involves costly maintenance of experimental set-ups and of the fish in their maintenance aquaria. The method described here will not only simplify laboratory studies, but also reduce costs and help to draw conclusions on growth rates of delicate organisms in the field, at remote places or on ships within a few hours without using hazardous radioactive material that are difficult to obtain or prohibited to use in some areas. It will also allow the determination of the fractional cost of protein synthesis in the context of the energy budget, e.g., [38]. Energy use plays an important role in setting tolerance to environmental changes, such as those caused by climate change. The proposed technique may help to perform such experiments more easily.

## Figures and Tables

**Table 1:** Time-dependent protein synthesis rates Ks and percentage of labeled phenylalanine in the free pool in white muscle tissue of *Pachycara brachycephalum* kept at 0 °C.

Time (h)	Ks (% Day^−1^)	Labeled, Free Phe (%)
1.5	0.052 ± 0.029	68.54 ± 12.36
3	0.055 ± 0.015	75.58 ± 14.14
4.5	0.041 ± 0.015	80.64 ± 5.57
Total Mean	0.049 ± 0.021	75.50 ± 11.07

## Data Availability

Data will be uploaded to the public repository PANGEA after acceptance of the manuscript.
